# Evaluation of Antioxidant Activity and Drug Delivery Potential of Cell-Derived Extracellular Vesicles from *Citrus reticulata* Blanco cv. ‘Dahongpao’

**DOI:** 10.3390/antiox12091706

**Published:** 2023-09-01

**Authors:** Shunjie Li, Zimao Ye, Lintao Zhao, Yijun Yao, Zhiqin Zhou

**Affiliations:** 1 College of Horticulture and Landscape Architecture, Southwest University, Chongqing 400716, China; lishunjie123@email.swu.edu.cn (S.L.); yezimao@email.swu.edu.cn (Z.Y.); 2 Key Laboratory of Agricultural Biosafety and Green Production of Upper Yangtze River (Ministry of Education), College of Horticulture and Landscape Architecture, Southwest University, Chongqing 400715, China; zlt19991116@email.swu.edu.cn; 3 The Southwest Institute of Fruits Nutrition, Banan District, Chongqing 400054, China; swuyibao@email.swu.edu.cn

**Keywords:** *Citrus reticulata* Blanco cv. ‘Dahongpao’, extracellular vesicles, antioxidant, drug delivery

## Abstract

Plant extracellular vesicles (PEVs) have attracted increasing attention due to their rich composition, good antioxidant and anti-inflammatory activity, and ability to transport drugs. As a common fruit, citrus is an ideal material for extracting PEVs because of the diversity and abundance of bioactive substances in it. In our study, citrus-derived extracellular vesicles (CEVs) were extracted from red mandarin (*Citrus reticulata* Blanco cv. ‘Dahongpao’) and it was found that they contain high levels of lipids, proteins, and carbohydrates. The high levels of total phenols and total flavonoids suggest that CEVs have good chemical antioxidant properties. We also demonstrated through cell experiments that CEVs have significant antioxidant and anti-inflammatory effects. Furthermore, we found that CEVs have an encapsulation rate of 71.5 ± 0.19% and a high drug-carrying capacity of 4.96 ± 0.22% and can enhance antioxidant and anti-inflammatory activity when loaded with tangeretin. Our results show that CEVs contain abundant bioactive components, have low toxicity, exhibit good antioxidant and anti-inflammatory properties, and can serve as drug delivery agents. This study has important implications for utilizing citrus materials and developing natural anti-oxidative and anti-inflammatory biomaterials.

## 1. Introduction

Extracellular vesicles (EVs) represent a diverse population of membrane-bound structures that are released by eukaryotic cells into the extracellular environment [1,2]. The diameter of these vesicles is typically below 500 nanometers, and they feature a phospholipid bilayer and encapsulate a variety of molecules including lipids, proteins, and nucleic acids [3,4,5]. Having traditionally regarded them as focal points for cellular waste elimination, recent studies have unveiled the multifaceted beneficial roles of extracellular vesicles in cellular signaling, antiviral defense, and various other physiological processes [6,7,8,9]. This discovery has sparked an enthusiastic exploration into the potential health benefits associated with extracellular vesicles.

Plant-derived extracellular vesicles (PEVs) are small vesicles, enclosed by a membrane, that are released by plant cells. These vesicles closely resemble the size and morphology of exosomes found in mammalian cells [10,11]. PEVs are considered fundamental components of the defense mechanism plants employ against pathogen invasion [12]. Alongside lipids, proteins, and nucleic acids, PEVs possess a rich assortment of metabolic byproducts within their internal compositions [13,14]. These metabolic byproducts often possess remarkable biological activities such as anti-inflammatory and antioxidant effects. The types and quantities of these metabolites are usually correlated with the extraction matrix of PEVs [13]. Thus, the inherent nutritional characteristics and potential health value of fruits have sparked significant interest in extracting PEVs from fruits for their antioxidant properties and potential application in nutritional therapies [15,16,17]. This growing interest stems from the recognition of the valuable role that PEVs derived from fruits can play in promoting overall health and well-being.

PEVs extracted from fruits such as grapes, grapefruits, lemons, apples, and strawberries exhibit notable antioxidant activity and contribute to maintaining intestinal homeostasis [16,18,19,20]. Furthermore, the ability of PEVs to withstand the challenges of the gastrointestinal environment has led to their recognition as the components of a novel drug delivery system [21,22]. By encapsulating exogenous molecules such as medications or other beneficial substances, PEVs offer an enhanced platform for delivering therapeutic agents with improved efficacy [23,24]. This unique characteristic of PEVs presents exciting opportunities for targeted and efficient drug delivery, opening new avenues in medical research and treatment.

The aim of this study is to extract extracellular vesicles from the red mandarin (*Citrus reticulata* Blanco cv. ‘Dahongpao’), analyze their composition and properties, investigate their potential role in mitigating biological cellular oxidative stress and inflammation, and assess their potential as carriers for drug delivery. To achieve this, the study used PEG precipitation to extract CEVs and was divided into three parts: 1. Characterization, composition analysis, chemical antioxidant activity testing, and storage stability testing of CEVs. 2. Studying the ability of CEVs to mitigate oxidative stress induced by H_2_O_2_ treatment and reduce LPS-induced cell inflammation in RAW264.7 macrophages. 3. The loading of tangeretin onto CEVs as a carrier and testing the antioxidant and anti-inflammatory effects of CEVs-TAN (Citrus-derived extracellular vesicles loaded with Tangeretin) on cells treated with H_2_O_2_ and LPS.

## 2. Materials and Methods

### 2.1. Reagents and Materials

Red mandarin *(Citrus reticulata* Blanco cv. ‘Dahongpao’) was gathered from Puya Village, Dazhou Town in Wanzhou District, Chongqing. It was then transported to the Key Laboratory of Agricultural Biosafety and Green Production at the School of Horticulture and Landscape Architecture, Southwest University in Chongqing, China. Tangeretin was procured from Shaanxi Huike Botanical Development Co., Ltd. (Shaanxi, China). PEG6000 was purchased from Chengdu Kelong Chemical Reagent Factory (Chengdu, China). PLGA was purchased from Nanjing Herbal Source Biotechnology Co., Ltd. (Nanjing, China). RAW264.7 mouse macrophages were purchased from Procell (Wuhan, China). Dulbecco’s Modified Eagle Medium (DMEM) was purchased from gibco(Shanghai, China). Fetal bovine serum (FBS) was purchased from Yeasen Biotechnology (Shanghai) Co., Ltd. (Shanghai, China). Penicillin/Streptomycin was purchased from Yeasen Biotechnology (Shanghai) Co., Ltd. (Shanghai, China). Lipopolysaccharides (LPSs) were purchased from Nanjing Herbal Biotechnology Co., Ltd. (Nanjing, China). Hydrogen peroxide (H202) was purchased from Chongqing Chuandong Chemical Co., Ltd. (Chongqing, China). Nitric oxide test kits were purchased in Beyotime (Shanghai, China). Superoxide dismutase kit, peroxidase kit, and peroxidase kit were purchased from Shanghai Youxuan Biotechnology Co., Ltd. (Shanghai, China). Polyvinyl alcohol (PVA) was purchased from Chengdu Kelogne Chemical Co., Ltd. (Chengdu, China).

### 2.2. Extraction of CEVs

Juice the whole fruit with a joyoung wallbreaker (model: L18-P161), then crush and homogenize. Filter the resulting homogenate through a 60-mesh, four-layer gauze to obtain a filtrate. Centrifuge the filtrate at 4 °C and 2000× *g* for 10 min to remove large particle impurities and collect the supernatant. Then, centrifuge the filtrate at 4 °C and 6000× *g* for 20 min to remove the precipitate and collect the supernatant. Next, centrifuge the filtrate at 4 °C and 10,000× *g* for 30 min to remove impurities such as tissues, cell debris, and lipids and collect the supernatant. Lastly, centrifuge the supernatant at 10,000× *g* until no impurity particles settle and collect the clear supernatant. Further filter the centrifuged supernatant through a 0.45 μm filter to remove smaller impurities. To prepare the 40% PEG6000 solution, weigh and dissolve 40 g of PEG6000 in 100 mL of ultrapure water. Then, mix the collected supernatant with the prepared solution to obtain a 10% PEG6000 concentration. Stir the mixture well and incubate it overnight at 4 °C. After incubation, centrifuge the mixture at 10,000× *g* for 30 min at 4 °C to form a precipitate. Dissolve the precipitate in an appropriate amount of PBS solution and filter it through a 0.22 µm filter to obtain CEVs using the PEG method.

### 2.3. Evaluation of CEVs

#### 2.3.1. Analysis of CEV Morphology by Using Transmission Electron Microscopy

Prepare samples for JEM-1200EX imaging (100 KV): place sealing film on glass sheet and secure copper mesh with forceps. Add 10 µL drops of sample to mesh and remove excess liquid with filter paper. Apply 10 µL of 3% uranyl acetate, wait 1–3 min, and remove excess stain with filter paper. Let mesh dry naturally for 10 min. The TEM (Transmission Electron Microscope, JEM-1200EX, JEOL Ltd., Tokyo, Japan) has a point resolution of 0.14 nm. A carbon support membrane with a pore size of 230 mesh is used. The imaging software (4.6.0) employed is Olympus Image Share.

#### 2.3.2. Malvern Laser Particle Size Analyzer

The Nano ZS90 particle size and zeta potential analyzer, along with a Nano Zetasizer Model ZS dynamic light scatterometer, was used to analyze the particle size and potential of extracellular vesicles from *Citrus reticulata* Blanco cv. ‘Dahongpao’. The prepared samples were diluted 50 times with PBS before the determination. The particle size of *Citrus reticulata* Blanco cv. ‘Dahongpao’ extracellular vesicles was measured at 25 °C at an angle of 173° using a Nano Zetasizer Model ZS dynamic light scatterometer. The measurements were performed at a temperature of 25 °C. The average values were determined by averaging the results of three measurements for each sample, with 100 runs per measurement.

#### 2.3.3. Identification of CEVs Components

##### Protein Content Determination

A quantity of 1 mL of CEV sample was taken out under freezing conditions and added to 500 μL of RIPA buffer with protease inhibitor (RIPA: FMSP = 100:1). The EVs’ suspension was mixed with strong RIPA lysis buffer and shaken. The EVs were ice-bathed for 30 min, and during this time, the sample was repeatedly pipetted up and down using a pipette gun to ensure complete cell lysis. The supernatant was collected using centrifugation at 12,000× *g* for 5 min and used as the total protein solution. The total protein content of the supernatant was determined using a BCA protein assay. Plant extracellular vesicles are similar to those of animals, and reference is made to the quantification method using exosomes, using the proteins of CEVs as standards for subsequent experiments [25,26].

##### Lipid Content Determination

The total lipid content of the CEVs was determined using the Bligh and Dyer method with methanol/chloroform extraction [27]. Under frozen conditions, 1 mL of the EV sample was extracted by adding a 2:1 (*v/v*) mixture of methanol/chloroform (3 mL). Chloroform (1 mL) and ddH_2_O (1 mL) were added to the mixture and vortexed. The mixture was centrifuged at 2000× *g* for 10 min at 22 °C to obtain two phases (i.e., aqueous and organic phases). The upper organic phase was taken and the solvent was rotary-evaporated at 60 °C until dryness. The weight of total lipids was measured after weighing the dried samples.

##### Total Sugar Content Determination

Under frozen conditions, 1 mL of CEV sample was taken out and added to 20 mL of anhydrous ethanol, and this was followed by ultrasound treatment for 30 min. The mixture was centrifuged at room temperature at 4000 r/min for 10 min, and the solvent fraction was removed. The remaining organic solvent was evaporated under nitrogen protection, then the weight of the dry residue, which represents the total sugar content, was measured. 

##### Determination of Total Phenol and Total Flavonoid Content

The extracted CEVs were firstly deconstructed using a low-temperature ultrasonic extraction solution to disrupt the vesicle structure, with 60% power for 2 s on and 2 s off, lasting for 1 min. The resulting CEVs were named Ult-CEVs. Then, the CEVs were chemically deconstructed using TritonX-100 at a volume ratio of CEVs to TritonX-100 of 1:1000. The treated CEVs were placed at room temperature for 30 min and named Trx-CEVs. The treated CEVs were freeze-dried to dry solids and dissolved into 80% methanol, and then their total phenolic and total flavonoid contents were determined. Total phenolic determination: The total phenolic content of the CEVs was measured using the Folin-phenol method. Specifically, 0.3 mL of CEVs was mixed with 4 mL of distilled water and 400 μL of Folin–Ciocalteu reagent. After being kept in the dark for 5 min, 2 mL of Na_2_CO_3_ solution (5%) was added. The mixture was then kept in the dark for 60 min for a reaction to occur before the total phenolic content was measured using a spectrophotometer at 765 nm. Total flavonoid determination: A quantity of 0.5 mL of the CEV sample was mixed with 0.7 mL distilled water and 0.2 mL of 5% NaNO_2_, and the mixture was shaken and placed in the dark for 6 min. Then, 0.2 mL of 10% Al(NO_3_)_3_ was added and shaken and the mixture was again placed in the dark for 6 min. Next, 2 mL of 1 mol·L^−1^ NaOH was added, and the mixture was shaken and brought up to a total volume of 5 mL with 1.4 mL distilled water. The mixture was shaken well and placed in the dark for a further 15 min. Absorbance was measured at 500 nm.

##### Determination of Flavonoids

The extracted CEVs were freeze-dried into solids and assayed by HPLC. The freeze-dried samples were mixed with 90% ethanol by volume, sonicated at 300 W for 30 min, and centrifuged at 1000× *g* for 10 min to obtain the supernatant, which was then analyzed by HPLC with specific parameters as described by Zixiao Jiang in the extraction process of polymethoxyflavones from the sour orange (*Citrus aurantium* L.) [28].

#### 2.3.4. Chemical Evaluation of the Antioxidant Capacity of CEVs

We measured the chemical antioxidant properties of CEVs, Ult-CEVs, and Trx-CEVs by using three assays, namely ABTS radical scavenging activity, DPPH radical scavenging activity, and FRAP ferric ion reduction capacity, which were used to evaluate the chemical antioxidant properties of CEVs. To standardize our results, we used Trolox as a reference standard and expressed the findings in Trolox Equivalent (TE) per mL (μmol/mLTE). Prior to analysis, all samples were diluted five times with distilled water. For the ABTS assay, we mixed ABTS solution (7 mM) and K_2_S_2_O_4_ solution (140 mM) and then allowed them to react for 12–16 h before diluting the mixture with anhydrous ethanol to an absorbance value of 0.70 ± 0.02 at 734 nm. Then, we added 40 μL of each sample or Trolox solution to 3.90 mL of the diluted ABTS solution and incubated this under light-proof conditions for 10 min before reading the absorbance at 734 nm. For the DPPH assay, we added 100 μL of each sample or Trolox solution to 3.80 mL of DPPH solution (75 μmol/L), which was protected from light for 30 min, and then measured the mixture at 517 nm. In the FRAP assay, we prepared acetate buffer (0.3 mol/L, pH = 3.6), FeCl_3_ solution (20 mmol/L), and TPTZ working solution (10 mmol/L) using HCl (40 mmol/L). We mixed these solutions in a 10:1:1 ratio to obtain TPTZ solution and then measured the absorbance at 593 nm after incubating 100 μL of each sample or Trolox solution with 3.90 mL of TPTZ solution for 30 min under light-proof conditions.

#### 2.3.5. Storage Stability

The CEVs was separately placed in centrifuge tubes and stored at two different temperatures, namely 4 °C and −80 °C. The stored samples were tested for changes in particle size and potential on days 1, 5, 10, and 15.

### 2.4. Assessment of Anti-Inflammatory and Antioxidant Capacity

#### 2.4.1. Cell Recovery and Culture

To prepare the complete medium, a combination of 10% serum and 1% penicillin and streptomycin in DMSO was used. The frozen RAW264.7 macrophages were then removed and thawed in a stable aqueous solution at 37 °C. After warming to 37 °C, the RAW264.7 macrophages were placed into a sterilized 50 mL centrifuge tube, and then, 4 mL of complete culture medium was added. Following this, the cells were centrifuged for 5 min at room temperature with a speed of 1000 rpm. Next, the supernatant was discarded and 4 mL of complete culture medium was added to resuspend the RAW264.7 macrophages. The suspension obtained was slowly added dropwise to a 6-well plate for incubation with light shaking to evenly spread the cells in the well (2 mL of cell suspension per well). After observation under a microscope, the RAW264.7 macrophages were subsequently incubated in an incubator at RH = 80%, 37 °C, and 5% CO_2_.

#### 2.4.2. Cell Viability Assay

An amount of 2 × 10^5^ cells in a volume of 100 μL was added to each well of a 96-well cell culture plate and incubated for 24 h. Following that, RAW264.7 cells were treated with fresh medium containing varying concentrations of CEVs and CEVs-TAN (ranging from 0 μg/mL to 400 μg/mL). After being placed in the incubator for another 24 h, the cell cultures were removed, and 10 µL of CCK-8 solution was added to each well. The plate was then incubated at 37 °C for 1 h before we measured the optical density (OD) at 450 nm using an enzyme marker from Thermo Scientific, Waltham, MA, USA.

#### 2.4.3. H_2_O_2_-Induced Oxidative Stress

The 30% H_2_O_2_ solution was diluted with complete medium to yield concentrations of 0.002–0.0055%. RAW264.7 macrophages were cultured in a 96-well plate and added with 100 μL of the prepared H_2_O_2_ solutions at different concentrations, with three replicates for each concentration treatment. The cell culture plates were then incubated for 2 h. After the incubation, the complete medium and H_2_O_2_ solutions in the plate were removed. A quantity of 100 μL of PBS was added slowly to wash the cells. Then, the PBS was aspirated, and a mixture of 100 μL of complete medium and CCK-8 reagent solution (in a ratio of 9:1) was added to each well. The plate was put back into the incubator for an additional hour. At the end of the incubation, the optical density (OD) of the cell culture medium at 450 nm was measured using a microplate reader.

#### 2.4.4. Nitrogen Monoxide (NO) Determination

After allowing the GriessA and B solutions to reach room temperature, transfer 50 μL of supernatant from each well into a new 96-well plate. Next, mix GriessA and B solutions in equal volumes and then add 100 μL of the mixed solution to each well of the 96-well plate containing the supernatant samples. Mix the contents of each well thoroughly before measuring the absorbance at 540 nm.

#### 2.4.5. Malondialdehyde (MDA) Determination

To perform the assay, 5 million collected cells were added to 1 mL of extraction solution. After cell lysis according to the kit instructions, 300 µL of Reagent 1 was added together with 100 µL of sample solution. The mixture was then subjected to centrifugation after a water bath treatment at 95 °C for 30 min. Next, 200 µL of supernatant was carefully transferred to a new 96-well plate for absorbance analysis. The absorbance values were measured at 532 nm and 600 nm.

#### 2.4.6. Antioxidant Enzyme Index Determination

The cells were collected and resuspended in 500 µL of extraction solution. Sonication was performed with a power of 20%, using 3-s pulses at 10-s intervals, for a total of 30 repetitions to break up the cells. The activity of CAT enzyme was measured by determining OD values at 240 nm, while POD enzyme activity was determined at 450 nm following the operating procedures provided in the respective kits. The OD values of CAT at 240 nm and POD at 450 nm were then recorded. For T-AOC determination, the cells were resuspended in 1 mL of extraction solution and sonicated using a power of 20% with 3-s pulses at 10-s intervals, repeated 30 times, as per the instructions of the Total Antioxidant Capacity Reagent kit. The OD value at 593 nm was determined to assess the total antioxidant capacity of the sample.

#### 2.4.7. LPS Induce Cellular Inflammation

To prepare a lipopolysaccharide (LPS) solution, we dissolved 1 mg of LPSs in 1 mL of PBS. The LPS solution was then diluted with complete medium to obtain a concentration of 3 μg/mL. Next, 5 × 10^5^ cells were added into each well of a six-well cell culture plate and incubated for 24 h until adherent. Following this, the supernatant was discarded, and 2 mL of 3 μg/mL LPS solution was added into each well. The cells were allowed to incubate with LPSs for 4 h.

#### 2.4.8. Expression Measurement of Inflammatory Factors

Cells were collected with a cell scraper and added to 600 µL of cell lysate (TIANGEN, Beijing, China). The lysed cells were used for RNA extraction and cDNA transcription using the Evo M-MLV Reverse Transcription Premix Kit (AG, Hunan, China). Quantification was performed using the SYBR Green Pro Taq HS Premix qPCR kit (AG, Hunan, China). Primers for inflammatory genes were synthesised by UW Genetics (Shenzhen, China).

### 2.5. CEVs Loaded with Tangeretin

#### 2.5.1. Preparation of CEVs-TAN

First, 0.1 g of PLGA and 0.1 g of Tangeretin were added to dichloromethane and sonicated at 60% power for 10 min until completely dissolved, then they were added to 1% PVA with constant magnetic stirring and placed on ice using a 20% power ultrasonic extractor working for 2 s and stopping for 2 s for a total of 2 min. At a temperature of 4 °C, we centrifuged the mixture at a speed of 8000 rpm for 10 min, removed the supernatant, and then centrifuged again for another 10 min at the same speed of 8000 rpm. The centrifuged supernatant was centrifuged at 14,000 rpm for 40 min at 4 °C, the supernatant was discarded, and the precipitate was taken and re-dissolved in 0.5 mL of PBS and collected to obtain PLGA-Tangeretin nanoparticles. The prepared CEVs were then mixed with PLGA-Tangeretin nanoparticles at a volume ratio of 1:1 and sonicated at 40% power for 5 min, followed by centrifugation at 14,000 rpm for 40 min at 4 °C. The supernatant was discarded and re-dissolved in PBS to obtain CEVs-TAN.

#### 2.5.2. Drug Encapsulation and Loading Efficiency Evaluation

The drug loading efficiency of CEVs and the encapsulation rate of Tangeretin by PLGA was determined by using a Beckman Coulter UV spectrophotometer. After being freeze-dried, the sample was weighed (10 mg) and then dissolved in a solution of ethanol:dichloromethane (10:1, *v*:*v*) at a concentration of 5 mL. The resulting mixture was sonicated for 10 min at 60% power, and subsequent to centrifugation at 8000× *g* for 10 min, the supernatant was collected for further determination. The UV spectrophotometer was employed to determine the maximum absorption wavelength of Tangeretin. The absorbance values at this wavelength were measured for different Tangeretin concentrations, and a standard curve was subsequently established. The encapsulation rate (ER) and loading capacity (LC) are calculated using the following equations:

(1) $$Encapsulation\;rate\;\left(ER\right)=\frac{1-free\;content}{input\;amount}$$

(2) $$Loading\;capacity\;\left(LC\right)=\frac{Extracellular\;vesicle\;drug\;content}{Total\;mass\;of\;extracellular\;vesicles}$$

### 2.6. Statistical Analysis

All data were reported as “mean ± standard deviation” of at least triplicate measurements per sample. One-way analysis of variance (ANOVA) and Duncan’s test were conducted using SPSS 17.0, and *p* < 0.05 was considered statistically significant.

## 3. Results

### 3.1. Identifification and Characterization of CEVs 

**Figure 1:**
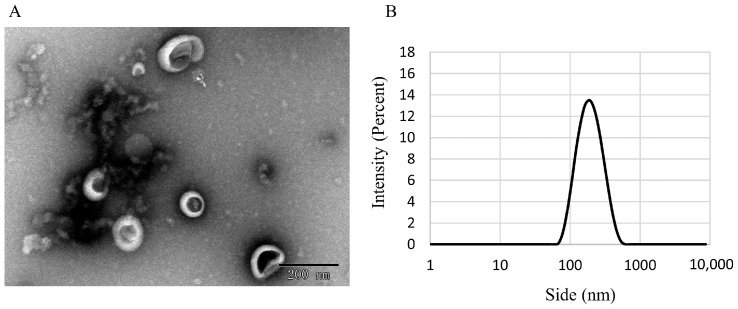
Shape and particle size of CEVs. Transmission electron microscopy morphology of CEVs (**A**); particle size diagram of CEVs (**B**).

This experiment described that the CEVs were obtained from juice by using the PEG6000 polymer precipitation method, as shown in Figure 1A. The transmission electron microscopy image shows that the extracted CEVs are spherical vesicles of about 200 nanometers in size, with irregular shapes in the middle of the vesicles, which is consistent with the experimental results of a previous study [29]. Figure 1B confirms the size range of CEV particles to be approximately 60–500 nanometers through the analysis of the nanometer particle characteristics and zeta potential analyzer, with an average size of about 190 nanometers. In addition, the measured electric potential is approximately −5 Mv, and the PDI coefficient is approximately 0.17, indicating that the distribution of CEVs is relatively narrow and has good singularity.

### 3.2. Chemical Composition of CEVs

**Figure 2:**
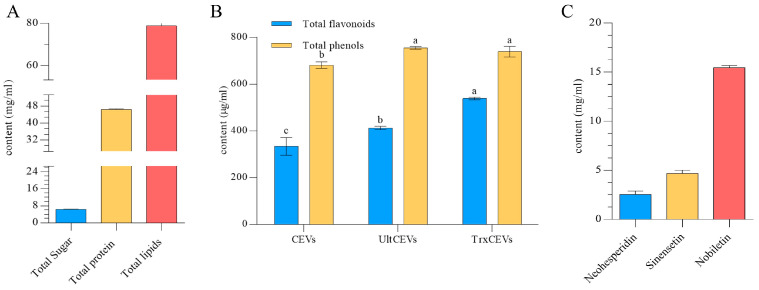
Determination of the chemical composition contents of CEVs. Determination of total sugar content, total lipids, and total protein content of CEVs (**A**); the total phenol and total flavonoid contents in CEVs were determined both by themselves as well as by ultrasonic deconstruction and chemical deconstruction (**B**); types and contents of flavonoids detected in CEVs (**C**). Values in the same row with different superscript letters are significantly different (*p* < 0.05).

Figure 2A displays the sugar, protein, and lipid contents of CEVs. The total sugar content of CEVs was determined to be 6.29 ± 0.18 mg/mL using the Phenol–Sulfuric Acid Assay method; the protein content was determined to be 46.38 ± 0.43 mg/mL using the BCA method, and the lipid content of CEVs was determined to be 78.80 ± 1.83 mg/mL using the Bligh and Dyer method.

Figure 2B determines the differences between the contents of total phenols and total flavonoids in CEVs under different conditions. The total phenol content in CEVs was 681.02 ± 13.98 μg/mL and the total flavonoid content was 333.61 ± 38.16 μg/mL. After ultrasonic deconstruction, the total phenol content in Ult-CEVs increased to 755.09 ± 5.38 μg/mL while the chemical deconstruction of Trx-CEVs revealed a total flavonoid content of 538.06 ± 5.36 μg/mL. The contents of both total phenols and total flavonoids after deconstruction were higher than those in CEVs. As shown in Figure 2C, three citrus flavonoids were detected in high quantities in the CEVs, consisting of Neohesperidin, Sinensetin, and Nobiletin, with the highest content being that of Nobiletin, reaching 15.44 ± 0.194 mg/mL, followed by Sinensetin at 4.678 ± 0.276 mg/mL. The Neohesperidin content was 2.54 ± 0.286 mg/mL.

### 3.3. Chemical Antioxidant Activity of CEVs

The CEVs, Ult-CEVs, and Trx-CEVs were analyzed for their antioxidant activity using ABTS, DPPH, and FRAP assays, respectively. The antioxidant capacity of the CEV samples was measured by eliminating ABTS+ and DPPH+ radicals and calculating the absorbance values. The reduction capacity was determined through a blue complex formation using the FRAP method. The chemical antioxidant capacity of the Ult-CEVs was found to be stronger than that of the CEVs, possibly due to the increase in antioxidant substances after ultrasonic deconstruction, leading to an enhancement of their antioxidant capacity (Table 1).

### 3.4. Storage Stability

Batches of prepared CEVs were stored at 4 °C and −80 °C to investigate variations in particle size during storage (Table 2). We measured the particle size, potential, and PDI indices of CEVs to observe their stability under different storage conditions. Over time, the particle size, potential, and PDI values of the CEVs stored at 4 °C exhibited an increasing trend. The particle size of the CEVs increased significantly from 165.63 ± 1.97 nm to 333.70 ± 11.72 nm during storage at 4 °C, with the particle size values more than doubling after 15 days of storage. In contrast, the particle size of the CEVs increased slightly from 172.90 ± 1.32 nm to 196.16 ± 1.69 nm when stored at −80 °C, and the increase in particle size over fifteen days was only around 23 nm. The potential of the CEVs decreased significantly from −5.38 ± 0.03 mV to −11.00 ± 0.46 mV, and the PDI increased from 0.17 ± 0.01 to 0.66 ± 0.10 after being stored for 15 days at 4 °C, indicating a decrease in the stability of CEVs during storage. Conversely, there was no substantial change observed in the potential and PDI of the CEVs stored at −80 °C from day one up to 15 days.

### 3.5. Effect of CEVs on Cell Viability

**Figure 3:**
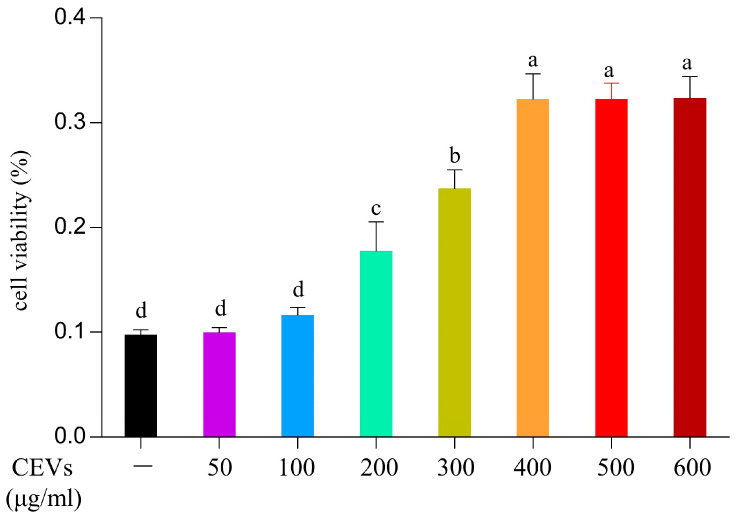
Toxicity of CEVs to RAW264.7 cells. Toxicity of different concentrations of CEVs on RAW264.7 cells. Values in the same row with different superscript letters are significantly different (*p* < 0.05).

RAW264.7 cells were treated with different concentrations of CEVs and incubated for 24 h in the cell culture box to detect their toxicity towards the cells. Cell viability was measured using a CCK-8 assay kit, and the corresponding values were calculated. The results showed that after 24 h of cultivation, the blank group’s cell viability was 79.40 ± 2.78% while the treatment group’s cell viability increased with an increase in the concentration of CEVs (Figure 3). When the CEVs reached a concentration of 400 μg/mL, cell activity reached 102.10 ± 3.87%, and increasing the CEV concentration to 500 μg/mL or 600 μg/mL showed no significant difference in cell activity compared to that of the group treated with 400 μg/mL, displaying an effect of promoting cell viability.

### 3.6. Cellular Antioxidant Activity of CEVs

#### 3.6.1. Determination of NO and MDA Indicators

**Figure 4:**
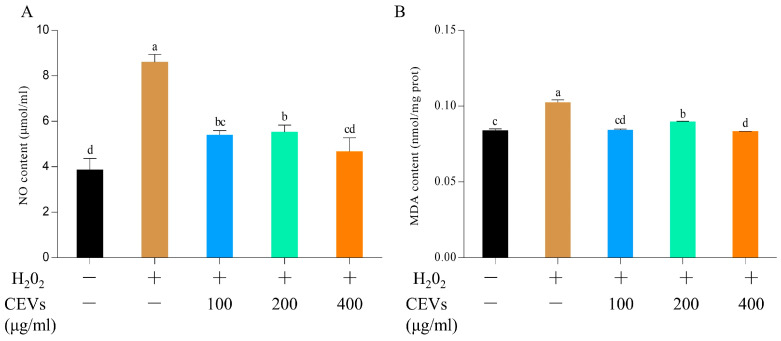
Determination of the effect of CEVs on cellular NO and MDA indicators after H_2_O_2_ treatment of cells. NO content in cell supernatants at different concentrations of CEVs (**A**). Effect of CEVs on changes in intracellular MDA content after H_2_O_2_ treatment (**B**). Note: ‘pro’ stands for protein. Values in the same row with different superscript letters are significantly different (*p* < 0.05).

Cells produced an excessive amount of NO when induced by hydrogen peroxide, which could cause damage to cells. The NO content in the cell culture medium was measured using an NO detection kit. As shown in Figure 4A, the control group’s NO concentration reached 8.6 ± 0.35 μmol/mL after hydrogen peroxide treatment. However, after adding CEVs, the NO content decreased in the cell culture medium. When the CEV concentration reached 400 μg/mL, the NO content in the cell culture medium decreased to 5.20 ± 4.67 μmol/mL and there was no significant difference compared to that of the blank group.

The MDA content in the cells was measured using an MDA detection kit to evaluate changes in the cells’ antioxidant capacity under different treatment conditions. As shown in Figure 4B, the blank group had an MDA content of 0.084 ± 0.0013 nmol/mg prot. Treatment with H_2_O_2_ led to an increase in the MDA content, which rose up to 0.1024 ± 0.0017 nmol/mg prot in the control group. However, after CEV treatment, the MDA content in the cells decreased. When the CEV concentration was 100 mg/mL, the MDA concentration in the cells was 0.085 ± 0.0007 nmol/mg prot, which did not differ significantly from the MDA content of the blank group. This indicates that CEVs have a certain antioxidant effect and can reduce the damage caused by oxidative stress to cells.

#### 3.6.2. Determination of Enzyme Activity Indicators

**Figure 5:**
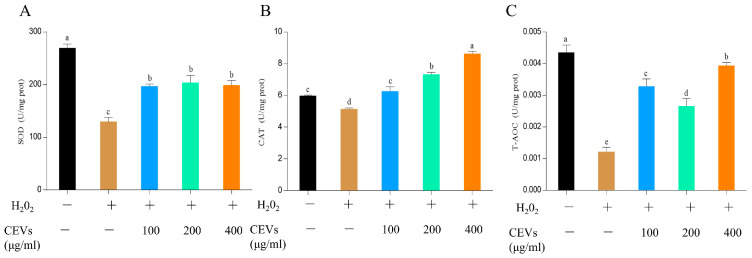
Determination of intracellular enzyme activity under different treatments of H_2_O_2_-induced oxidative stress in cells. SOD content and differences under different CEV concentration treatments (**A**); CAT content and differences under different CEV concentration treatments (**B**); T-AOC content and differences under different CEV concentration treatments (**C**). Values in the same row with different superscript letters are significantly different (*p* < 0.05).

This study aimed to evaluate the antioxidant capacity of RAW264.7 cells treated with different concentrations of CEVs and CEVs-TAN after H_2_O_2_-induced oxidative stress by analyzing SOD, CAT, and T-AOC assay kits. The results are shown in Figure 5A; following H_2_O_2_ treatment, the SOD value of the cells decreased significantly from 269.72 ± 7.49 U/mg prot to 129.74 ± 7.84 U/mg protein. However, CEV treatment increased the SOD value, which reached 198.61 ± 9.34 U/mg prot at a concentration of 400 μg/mL, similar to that of 100 μg/mL, suggesting that CEVs can enhance cellular SOD activity. As shown in Figure 5B, after H_2_O_2_ treatment, the CAT value in the cells was 5.14 ± 0.083 U/mg prot. Treatment with CEVs at a concentration of 100 μg/mL resulted in a CAT value of 6.27 ± 0.28 U/mg prot, similar to that of the blank group, but when the concentration was increased to 400 μg/mL, a significant increase in the CAT value was observed; it measured 8.63 ± 0.15 U/mg prot, indicating CEVs’ ability to promote cellular antioxidant capacity. These findings suggest that CEVs can elevate cellular CAT content and strengthen cellular antioxidant defense mechanisms. Moreover, the normal T-AOC value in the cells was 0.0044 ± 0.00024 U/mg prot, which decreased to 0.0012 ± 0.00014 U/mg prot after H_2_O_2_ treatment. However, CEV treatment restored the T-AOC values gradually, bringing them to 0.0039 ± 0.00011 U/mg prot at a concentration of 400 μg/mL, indicating CEVs’ ability to promote the recovery of the cellular antioxidant capacity after oxidative stress, as shown in Figure 5C.

### 3.7. Anti-Inflammatory Activity of CEVs

#### 3.7.1. Determination of NO Indicators

**Figure 6:**
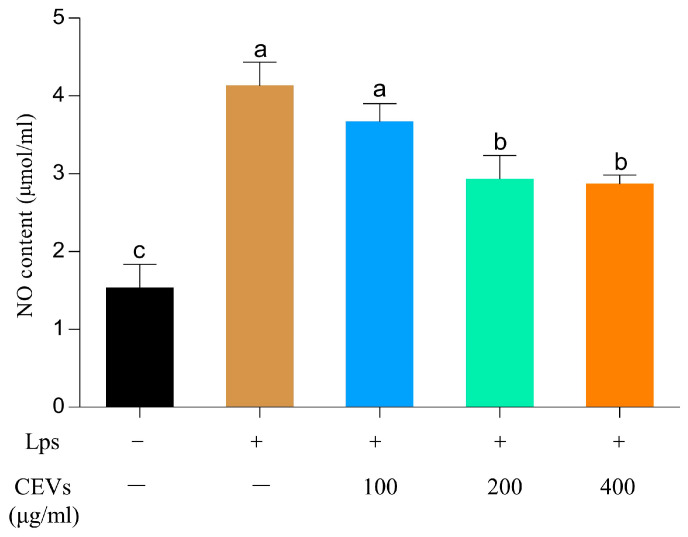
The content of cellular NO under different CEV concentration treatments after LPS induction. Values in the same row with different superscript letters are significantly different (*p* < 0.05).

The study induced inflammation in cells using 3 μg/mL of LPSs and assessed the cellular status under different concentrations of CEV treatment by determining the NO levels. As shown in Figure 6, the NO content of cells in the blank group was 1.53 ± 0.31 μmol/mL. After LPS treatment, the NO content increased to 4.13 ± 0.31 μmol/mL, indicating a significant 2.82-fold increase compared to the blank group. However, treating cells with CEVs gradually reduced the NO content as the CEV concentration increased. When the concentration of CEVs reached 400 μg/mL, the NO content in cells was restored to 2.87 ± 0.12 μmol/mL. The experimental results demonstrate that LPS treatment induces cellular inflammation and significantly increases cellular NO content. However, treating cells with different concentrations of CEVs effectively reduces the cellular NO levels. The restoration of NO content to near-normal levels at a CEV concentration of 400 μg/mL suggests that CEVs can effectively reduce the elevation of NO content caused by inflammation in cells.

#### 3.7.2. Measurement of Inflammatory Factors

**Figure 7:**
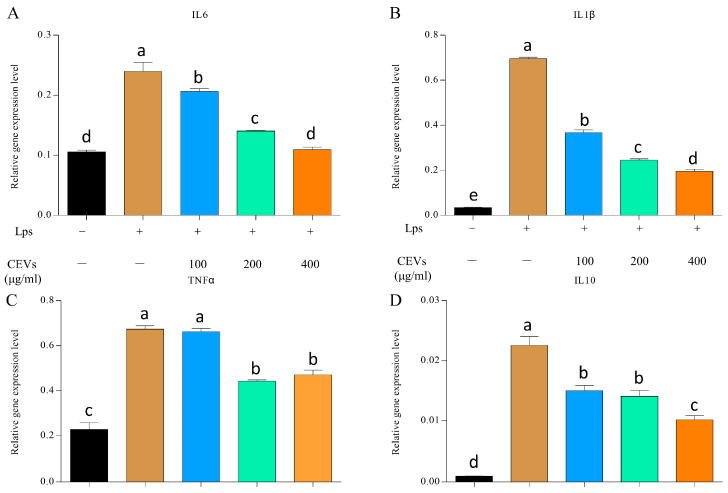
Differences between the expressions of inflammatory factors under different concentrations of CEV treatment after LPS-induced cellular inflammation. Differences between *IL-6* expression under different concentrations of CEV treatment (**A**); differences between *IL-1β* expression under treatment with different concentrations of CEVs (**B**); differences between the expression of *TNFα* under different concentrations of CEV treatment (**C**); differences between *IL-10* expression under different concentrations of CEV treatment (**D**). Values in the same row with different superscript letters are significantly different (*p* < 0.05).

The experiment determined the changes in the expression of pro-inflammatory factors (*IL-6*, *IL-1β*, *TNFα*) and anti-inflammatory factors (*IL-10*) under different concentrations of CEV treatment after LPS induction (Figure 7). After LPS induction, the gene expression levels of cellular pro-inflammatory factors (*IL-6*, *IL-1β*, and *TNFα*) were significantly increased compared to the blank group. The expression of *IL-6* increased from 0.106 ± 0.0027 in the blank group to 0.240 ± 0.0149. However, after CEV treatment, the expression of these pro-inflammatory factors decreased. LPS induction significantly increases the gene expression levels of pro-inflammatory factors (*IL-6*, *IL-1β*, and *TNFα*) in cells, while CEV treatment can effectively reduce their gene expression levels. At a concentration of 400 μg/mL CEVs, the expressions of *IL-6*, *IL-1β*, and *TNFα* decreased to 0.195 ± 0.00940, 0.470 ± 0.0213, and 0.195 ± 0.00940, respectively. At a CEV concentration of 400 μg/mL, the expressions of *IL-6*, *IL-1β*, and *TNFα* decreased to near-normal levels, indicating the potential therapeutic effect of CEVs against inflammation. After CEV treatment at a concentration of 400 μg/mL, the expression of *IL-10* decreased from 0.0226 ± 0.00148 in the control group to 0.0102 ± 0.00071.

### 3.8. Preparation of CEVs-TAN and Its Antioxidant and Anti-Inflammatory Evaluation

#### 3.8.1. Loading of Tangeretin by CEVs

**Figure 8:**
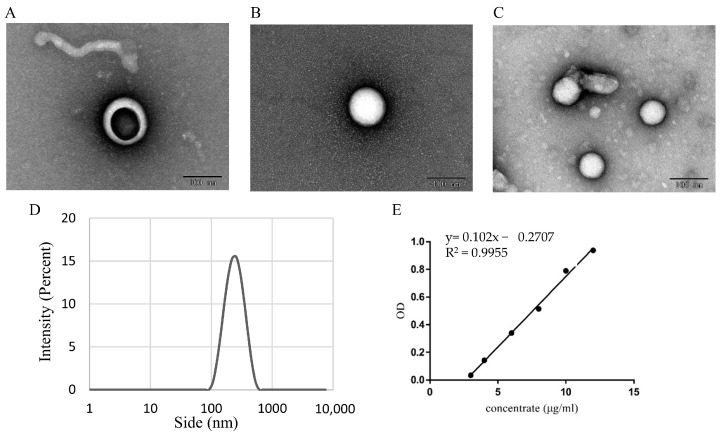
CEVs loading Tangeretin: indicator assay. CEVs (**A**), PLGA-TAN (**B**), and CEVs-TAN (**C**) projection electron microscope images; particle size diagram of CEVs-TAN (**D**); standard curve of Tangeretin (**E**).

CEVs were obtained from red mandarin by whole fruit juice extraction using the PEG6000 polymer precipitation method as shown in Figure 8A. The extracted CEVs were combined with PLGA-TAN nanoparticles in a volume ratio of 1:1:*v*:*v*, as shown in Figure 8B, and then ultrasound treatment was used to enable the CEVs to encapsulate the PLGA-TAN, resulting in CEVs-TAN, as shown in Figure 8C. It is clear from the TEM image that PLGA-TAN is encapsulated by CEVs and has a good dispersion. As shown in Figure 8D, the size range of the CEVs-TAN particles was determined by analysing the properties of the nanoparticles and the zeta potential analyser; the particle size range of CEVs-TAN was comparable to that of the CEVs, but the average size was approximately 220 nm. The measured potential was approximately −4.8 mV and the PDI coefficient was approximately 0.17.

The solubility of tangeretin was measured by dissolving it in a 10:1 mixture of ethanol and dichloromethane and then measuring the maximum wavelength of absorption using a UV spectrophotometer set at a wavelength of 320 nm. To construct a standard curve for the solubility of tangeretin, a sample of tangeretin monomer was diluted to concentrations in the range 3–12 μg/mL and the absorbance values were measured using UV spectrophotometry. The equation describing the linear relationship between concentration and absorbance for the solubility of tangeretin is y = 0.102x − 0.2707 (Figure 8E). This linear regression equation can be used to calculate the solubility of Tangeretin at any given concentration from its absorbance value. The amount of Tangeretin and PLGA precipitated after loading was determined by UV-Vis spectrophotometry. The amount of free tangeretin after centrifugation was then calculated using the same method. Using the formula given in Section 2.5.2, the encapsulation efficiency of CEVs-TAN for Tangeretin was calculated to be 71.5 ± 0.19%. The drug loading capacity of red mandarin extracellular vesicles for tangeretin was determined using UV spectroscopy and found to be 17.83 ± 0.63 mg/mL. Based on this formula, the total mass of CEVs (represented by the protein content) compared to the loaded Tangeretin content is the drug loading capacity of CEVs-TAN, which was calculated to be 4.96 ± 0.22%.

#### 3.8.2. Antioxidant and Anti-Inflammatory Evaluation of CEVs-TAN

In this study, the antioxidant capacity of CEVs-TAN after tangeretin loading was evaluated by comparing 400 μg/mL concentrations of CEVs, CEVs-TAN, and TAN with an equivalent dose to CEVs-TAN. The oxidative potential of CEVs-TAN was assessed by measuring MDA content, SOD, and T-AOC levels. The results demonstrated that the MDA content in the CEVs-TAN group was significantly lower, at 0.0636 ± 0.000485 nmol/mg, compared to the TAN and CEV groups. Moreover, both CEVs and CEVs-TAN treatment groups exhibited the highest levels of SOD and T-AOC, at 236.299 ± 9.773 U/mg prot and 0.00456 ± 0.000277 U/mg prot, respectively. These findings suggest that loading tangeretin onto CEVs can significantly enhance their antioxidant activity, and CEVs-TAN has a strong antioxidant capacity compared to TAN and CEVs.

**Figure 9:**
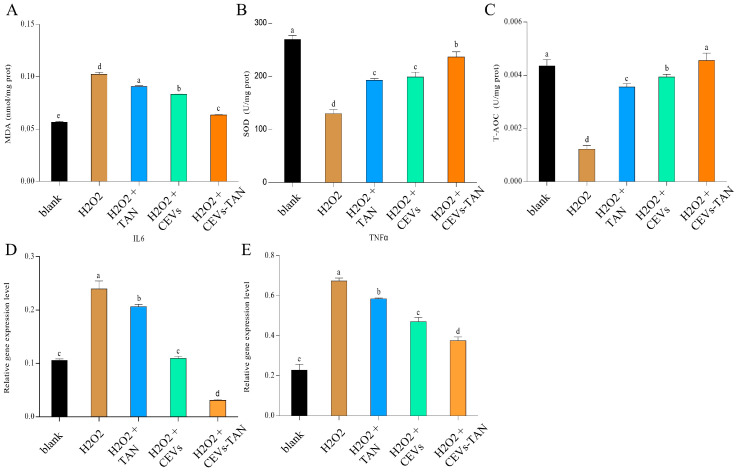
Determination of antioxidant indices and expression of anti-inflammatory factor levels in cells under CEVs-TAN treatment. Differences in MDA (**A**), SOD (**B**), and T-AOC (**C**) contents of cells treated with CEVs, TAN, and CEVs-TAN after hydrogen peroxide-induced oxidative stress; differences in *IL-6* (**D**) and *TNFα* (**E**) gene expression in cells treated with CEVs, TAN, and CEVs-TAN after LPS-induced cell inflammation. Values in the same row with different superscript letters are significantly different (*p* < 0.05).

In the LPS-induced inflammation model, the CEVs-TAN group showed significantly lower levels of *IL-6* and *TNFα* gene expression compared to the TAN and CEV treatment groups, with values of 0.0310 ± 0.00189 and 0.375 ± 0.0195, respectively (as shown in Figure 9D,E). Moreover, there were significant differences in the gene expression levels of *IL-6* and *TNFα* between the CEVs-TAN group and both the TAN and CEV treatment groups. In summary, these results suggest that the CEVs-TAN group may present a more effective approach for treating LPS-induced inflammation.

## 4. Discussion

Extracellular vesicles (EVs) play a critical role in mediating intercellular communication in various organisms, serving to maintain stem cells and regulate immune function, repair body tissues [30], and transport proteins, sugars, lipids, bioactive substances, and genetic material within their lipid bilayer packaging during physiological and pathological processes [31,32]. In the medical field, extracellular vesicles are used as a natural drug with an efficient loading effect, allowing the loading of multiple drugs, prevention of drug degradation, and enhanced endocytosis for the delivery of therapeutic agents or anticancer agents [33,34,35]. Existing studies have tested and evaluated the effects of extracellular vesicles loaded with proteins, small molecule drugs, nanoparticles, genetic material, etc. [36,37]. In the field of plant research, extracellular vesicles (EVs) extracted from plants have also been used as tools for drug delivery. EVs derived from tomato and grapefruit can be used as nanocarriers to load heat shock protein 70 (HSP70) and deliver it to glioblastoma cells [38]. In addition to their protein delivery capabilities, extracellular vesicle-like nanoparticles derived from cabbage exhibit the remarkable ability to encapsulate antisense DNA oligonucleotides and successfully transport them into human cells. This exciting finding highlights the significant promise of utilizing plant extracellular vesicles for advanced nucleic acid therapies [24]. The wide range of biological sources of plant-derived EVs, their good biological activity, and their potential as nano-delivery carriers have led to enthusiasm for research on EVs from different plant sources, especially edible ones.

A large number of extracellular vesicles (EVs) were isolated from whole fruit of red mandarin (*Citrus reticulata* Blanco cv. ‘Dahongpao’) using PEG sedimentation in this experiment. Firstly, the isolated EVs were analyzed and identified. Transmission electron microscopy demonstrated that the CEVs were spherical or irregularly spherical with a particle size of about 150 nm. These results suggest that we extracted small vesicles from red mandarin, as these vesicles usually have a particle size of less than 200 nm [39]. The morphology and particle size of our CEVs were similar to those of the vesicles obtained from grapefruit, but differed significantly from those obtained from ginger, which had a particle size of 300–400 nm [40,41]. This difference may be related to the fact that we used 0.22 μm filter membranes during the extraction of CEVs. We obtained the total sugar, total protein, and total lipid content of the extracted extracellular vesicle sample. As CEVs are composed of a phospholipid bilayer structure, they primarily consist of lipid components, with the measured lipid content at 78.80 ± 1.83 mg/mL being the highest among the measured components. Additionally, CEVs contain a wide range of protein components, with a protein content of 46.38 ± 0.43 mg/mL. EVs usually include a variety of metabolites as part of intercellular communication, and these metabolites generally possess favorable bioactivity and are some of the main components of EVs that perform their functions [42]. In addition to the presence of neohesperidin, we also detected the presence of polymethoxyflavones (PMFs) in the CEVs we obtained, wherein the PMFs components were noliletin and sinensetin. This may be related to the fact that we used red mandarin to extract the EVs in our experiments, as the CEVs contained components that were consistent with those we previously detected in red mandarin [43]. This finding of ours is also consistent with previous studies, such as those on grapefruit-derived EVs containing higher amounts of naringin, the most abundant flavonoid in grapefruit [44]. This again demonstrates the consistency between the components contained in EVs and their extracted materials. As a sub-group of plant flavonoids, PMFs have been found to possess broad biological activities such as anticancer, anti-inflammatory, antioxidant, neuroprotective, and antimicrobial effects, which have garnered increasing attention in the research community [45]. The experiment demonstrated that CEVs exhibit more significant antioxidant activity under chemical and physical disruption, which may be attributed to the enhanced antioxidant capacity resulting from the exposure of bioactive substances when the structure of red mandarin extracellular vesicles is damaged.

EVs from edible plant sources have shown promising results in health promotion, especially in antioxidant qualities and inflammation inhibition. For example, extracellular vesicles from strawberry have shown significant effects in preventing oxidative stress in human mesenchymal stromal cells [46]. On the other hand, ginger-derived extracellular nanoparticles can inhibit inflammation by attenuating the expression of inflammatory factors (*TNFα*, *IL-6*, *IL-1β*) in the lungs [47]. The results of our cell experiments revealed that CEVs can effectively alleviate H_2_O_2_-induced oxidative stress, as evidenced by measurements of NO, MAD, and oxidative stress-related enzyme activity. Furthermore, CEVs can also effectively reduce the inflammation response induced by LPSs. These findings provide important guidance and basis for the use of extracellular vesicles in the treatment of various diseases, which can be considered as emerging medical materials with wide application prospects. Extracellular vesicles have shown great potential for treating cardiovascular diseases, neurodegenerative diseases, inflammatory bowel disease, cancer, and other conditions by delivering specific bioactive substances such as proteins, nucleic acids, and drugs to target cells. Moreover, their wide availability and ease of cultivation and production, as well as their relative safety and low toxicity, make extracellular vesicles a feasible option for development and application as drugs or health supplements from both plant and animal sources. Therefore, extracellular vesicles can be seen as new and ideal medical materials with broad application potential [48].

The phospholipid layer contained in EVs and the inherent nanoscale size make EVs components of a drug delivery system of natural origin. It has been shown that EVs from grapefruit, cabbage, and ginseng can encapsulate specific bioactive molecules to exert their effects [23,24,38]. In addition to the direct application of EVs as drug delivery carriers, lipids can be extracted from EVs and further encapsulated with exogenous drugs to obtain lyophilised nanodrugs. For example, lipid components have been extracted from mulberry leaves and further encapsulated with FP127 to obtain nanomedicines for the treatment of colitis [49]. Lipids derived from grapefruit have been used to prepare synthetic nanoparticles to deliver short interfering RNA, DNA expression vectors, and proteins to different types of cells [27]. In this experiment, we found that CEVs have good anti-inflammatory and antioxidant properties and can be used as novel antioxidants, and in order to explore the abilities of CEVs as naturally sourced antioxidants even further, the possibility of using CEVs as nanocarriers for drug encapsulation was explored. From the electron microscopy images, it was revealed that after sonication, the CEVs encapsulated PLGA-TAN, showing good field-of-view cleaning and dispersion. It was also found that the particle size of the encapsulated CEVs-TAN was larger than that of the original CEVs, which is consistent with the findings of other groups [50]. Cytotoxicity tests showed that CEVs had no toxic effects on cells, highlighting their potential as good medical delivery materials due to their low toxicity, low-cost mass production, biodegradability, and absence of biological contamination [51]. It was also found that loading tangerine onto CEVs did not alter the bioactivity of tangeretin but exhibited good antioxidant and anti-inflammatory functions. This finding is consistent with previous findings where researchers used grapefruit-derived extracellular vesicles in combination with hydrophobic agents including curcumin, folic acid, and Zymosan A [27]. While the experiment has yielded promising results, it is clear that there is still much to be learned about plant cell vesicles. The composition of these vesicles is complex and contains a rich variety of genetic materials, including mRNA, miRNA, and other RNAs. Future research should focus on exploring the relationship between mRNA and the antioxidant and anti-inflammatory properties of red mandarin extracellular vesicles in greater depth [52].

## 5. Conclusions

In conclusion, CEVs were extracted from red mandarin fruit and analyzed using transmission electron microscopy and particle size measurements to confirm their origin as red mandarin extracellular vesicles. The components of CEVs were analyzed, revealing high levels of total sugars (6.29 ± 0.18 mg/mL), total proteins (46.38 ± 0.43 mg/mL), and lipids (78.80 ± 1.83 mg/mL). Total phenols and flavonoids were also detected in the CEVs after lysis, with concentrations of 755.09 ± 5.38 μg/mL and 538.06 ± 5.36 μg/mL, respectively. Three types of citrus flavonoids, including Sinensetin, naringin, and neohesperidin, were also detected in the CEVs. Comprehensive analysis of various indicators, such as particle size, potential, and PDI, revealed that CEVs stored at −80 °C were relatively stable. Regarding the exploration of CEVs’ functions and applications, chemical antioxidant assays showed that CEVs displayed good antioxidant activity. In cell experiments, CEVs exhibited a strong ability to reduce oxidative stress-related enzyme activity caused by H_2_O_2_, demonstrating excellent cellular antioxidant performance. Additionally, CEVs reduced the expression levels of inflammation factors (*IL-6*, *TNFα*, *IL-1β*, and *IL-10*) induced by LPSs, showing anti-inflammatory potential. Furthermore, Tangeretin was encapsulated into the CEVs at an encapsulation efficiency of 71.5 ± 0.19%, and the drug-loading capacity was 4.96 ± 0.22%. After encapsulation, CEVs-TAN demonstrated good antioxidant and anti-inflammatory abilities. In summary, the results of this study provide insights into both the composition and stability of CEVs extracted from red mandarin fruit as well as their biological activities and potential applications for drug delivery and disease treatment.

## Figures and Tables

**Table 1:** Chemical antioxidant capacity of CEVs under different conditions.

NO.		Antioxidant Activities (μmol/g TE)				
		ABTS	DPPH	FRAP	APC	Rank
1	CEVs	375.78 ± 1.92 ^b^	68.56 ± 0.22 ^c^	185.43 ± 0.58 ^c^	94.32	3
2	Ult-CEVs	369.11 ± 1.92 ^c^	75.62 ± 0.77 ^a^	192.60 ± 0.50 ^a^	98.11	1
3	Trx-CEVs	391.33 ± 0.00 ^a^	73.05 ± 0.22 ^b^	187.43 ± 0.29 ^b^	97.97	2

Note: ABTS, DPPH, and FRAP are methods used to measure antioxidant activity while APC represents the total score of all three measurements combined. The rank shows the order of each sample’s overall antioxidant capacity. Values in the same row with different superscript letters are significantly different (*p* < 0.05).

**Table 2:** Changes in physical characteristics of CEVs at different storage temperatures and times.

Simple Name	Storage Temperature (°C)	Storage Time (Day)	Average Particle Size (nm)	Zero Potential (mV)	PDI
CEVs	4	1	165.63 ± 1.97 d	−5.38 ± 0.03 d	0.17 ± 0.01 c
		5	185.93 ± 1.31 c	−6.54 ± 0.35 c	0.20 ± 0.01 c
		10	247.73 ± 6.00 b	−9.90 ± 0.17 b	0.52 ± 0.05 b
		15	333.70 ± 11.72 a	−11.00 ± 0.46 a	0.66 ± 0.10 a
	−80	1	172.90 ± 1.32 c	−4.46 ± 0.27 a	0.17 ± 0.02 a
		5	202.40 ± 3.86 a	−3.57 ± 0.42 a	0.18 ± 0.01 a
		10	200.77 ± 1.76 ab	−5.86 ± 0.83 b	0.20 ± 0.03 a
		15	196.16 ± 1.69 b	−4.13 ± 0.56 a	0.18 ± 0.02 a

Note: Values in the same row with different letters are significantly different (*p* < 0.05).

## Data Availability

Data are contained within the article.
